# Awareness of environmental legislation as a deterrent for wildlife crime: A case with Masaai pastoralists, poison use and the Kenya Wildlife Act

**DOI:** 10.1007/s13280-021-01695-8

**Published:** 2022-01-25

**Authors:** Zahra Didarali, Timothy Kuiper, Christiaan W. Brink, Ralph Buij, Munir Z. Virani, Eric O. Reson, Andrea Santangeli

**Affiliations:** 1 School of Geography and the Environment, University of Oxford, Oxford, UK; 2 Centre for Statistics in Ecology and Conservation, Department of Statistical Sciences, University of Cape Town, Cape Town, 7700 South Africa; 3 BirdLife South Africa, Isdell House, 17 Hume Road, Dunkeld West 2196, Johannesburg, South Africa; 4 FitzPatrick Institute of African Ornithology, DST-NRF Centre of Excellence, University of Cape Town, Cape Town, South Africa; 5 The Peregrine Fund, 5668 West Flying Hawk Lane, Boise, ID 83709 USA; 6 Wageningen University, Droevendaalsesteeg 3A, 6708 PB Wageningen, The Netherlands; 7 Mohamed Bin Zayed Raptor Conservation Fund, P.O. Box 129555, Abu Dhabi, UAE; 8 Research Centre for Ecological Change, Organismal and Evolutionary Biology Research Programme, University of Helsinki, 00014 Helsinki, Finland

**Keywords:** Biodiversity hotspot, Environmental law, Environmental crime, Poisoning, Vulture crisis

## Abstract

Illegal wildlife crime is a global phenomenon, accelerating the ongoing biodiversity crisis. In the Old World, and particularly in Africa, illegal use of poisons to eliminate carnivores is the main driver of the continental vulture crisis. Knowledge about the underlying source and drivers of this threat is lacking for most areas, including Kenya, a global vulture and biodiversity hotspot. An extensive questionnaire survey of over 1300 respondents was run, using a specialized questioning technique and quantitative analytical approaches. Results show that, while pastoralists have a positive attitude towards vultures, over 20% of them use poisons to eliminate predators. Poisoning was largely driven by livestock losses to predators, and by negative attitude towards predators. Poisoning was less prevalent among respondents aware of the Kenya Wildlife Act. Overall, we suggest that a combination of top-down, e.g. legislation, and bottom-up (such as corrals or compensation) along with awareness campaigns may help reduce poisoning on the ground.

**Supplementary Information:**

The online version contains supplementary material available at 10.1007/s13280-021-01695-8.

## Introduction

Conflicts between humans and wildlife represent an important conservation challenge in the Anthropocene (Redpath et al. 2013). Such conflicts are becoming ubiquitous wherever people share space with wildlife, particularly carnivores or megafauna (van Eeden et al. 2018; Treves and Santiago‐Ávila 2020). To better understand and mitigate human–wildlife conflicts, it is crucial to understand the human perspective, scan for solutions and quantify their potential effectiveness (Redpath et al. 2013).

In the recent decades, the field of conservation science has made great progress with regards to embracing and understanding the human dimension within socio-ecological systems (Bennett et al. 2017). For example, approaches to study sensitive behaviors, such as illegal practices, developed within the social science, have now become popular in conservation to robustly quantify and understand environmental crime (Nuno and St John 2015). These sensitive questioning approaches are furthering our knowledge of the prevalence and correlates of environmental crimes, such as illegal forest logging, hunting and trapping of wildlife, collection of endangered plants, and use of poisons to control predators, among others (Nuno et al. 2013; St John et al. 2015; Santangeli et al. 2016; Craig et al. 2019; Hinsley et al. 2019).

Among all solutions to mitigate human–wildlife conflicts, changing human behavior at the individual level is commonly recognized as a crucial component for successful conservation efforts (St John et al. 2013). Initiatives targeting individual behaviors typically rely on the premise that lack of awareness is a key cause of non-participation in pro-environmental behaviors (van der Ploeg et al. 2011). However, social and environmental psychology research suggests that awareness alone is insufficient in triggering behavioral change (Abrahamse et al. 2005). Environmental education and awareness campaigns may not suffice in fixing environmental problems. Broad-scale approaches, such as policy and legislation, may represent effective top-down means to promote pro-environmental practices and behaviors (Gray and Shimshack 2011; Moreto and Gau 2017). However, lack of enforcement and negligible sanction levels, particularly in the Global South, typically result in compliance being voluntary at most (Keane et al. 2008; Moreto and Gau 2017).

Across Africa, but also beyond, an escalating human–wildlife conflict is often tragically resolved on-the-ground by people attempting to poison the offending wildlife, typically carnivores. This practice, while illegal in most countries (Ogada et al. 2012; Ogada 2014), endangers not only the target species, but also non-target species, such as avian scavengers. Vultures are in fact among the most common and numerous unintended victims of poisoning practices in Africa (Ogada et al. 2016; Botha et al. 2017). Consequently, poisoning represents the most rampant and critical threat to vultures across Africa, already causing widespread declines in many vulture populations, at such a level that it has been defined as a continental vulture crisis (Ogada et al. 2016; Botha et al. 2017; Margalida et al. 2019).

This vulture crisis is acute in Kenya (Virani et al. 2011), which represents a global vulture priority area (Santangeli et al. 2019), supporting important populations of several endangered and critically endangered vultures (such as *Gyps rueppellii*, *Gyps africanus*, *Necrosyrtes monachus* and *Trigonoceps occipitalis*, among others). Human–wildlife conflicts are particularly frequent in Kenya because over 65% of the country’s wildlife are found outside of protected areas (Western et al. 2009), on private and communal lands typically used for pastoralism. Particularly in Southern Kenya, a global biodiversity hotspot, the conflict between Masaai pastoralists and carnivores has escalated in recent decades, largely owing to the fast human population growth (Ogutu et al. 2016), among other factors (see e.g. Mukeka et al. 2019). This conflict resulted in the increased use of poisons aimed to eliminate carnivores by pastoralists, further endangering the local and regional vulture populations (Virani et al. 2011). Moreover, such an increased use of poisons may have roots not only in the escalating human–wildlife conflict, but also in shifts in local Maasai attitudes towards wildlife (Fernandez-Llamazares et al. 2020) and in the more stringent protection of wildlife which could have promoted the use of more subtle and anonymous means to kill predators, such as poisoning.

Conservation efforts have been put in place to mitigate the consequences of poisoning carnivores, such as rapid response to poisoning incidences (Murn and Botha 2017). However, these rapid reactive interventions need to be accompanied by pro-active efforts, in order to mitigate the problem at its root, e.g. through strategies that would discourage and divert local pastoralists from using poisons as a mean to resolve human–wildlife conflict situations. To this end, the Wildlife Conservation and Management Act 2013 (No. 47 of 2013) of Kenya (hereafter Wildlife Act) was designed in response to escalating rhino and elephant poaching in Kenya, as well as widespread wildlife declines. This legal Act increases the sanctions for environmental crimes, such as poaching or poisoning of wildlife (Kaai et al. 2013), as well as formalizing principles of sustainable use of natural resources, equitable sharing of the benefits derived from nature, and compensation schemes, e.g. resulting from livestock depredation from carnivores. Increasing the severity of sanctions is typically assumed to reduce target crimes through a deterrent effect (Moreto and Gau 2017), but this assumption has not often been tested with respect to environmental crime.

In order for these strategies, like legislation or other mitigation measures, to be effectively designed and implemented, there is an urgent need to understand, characterize and quantify the problem and how the solutions are perceived, from the human perspective. Only by achieving a deep understanding of the underlying drivers of human behavior it will be possible to trigger a change. While human–wildlife conflict has received much research and conservation attention (e.g. Redpath et al. 2013), also in Southern Kenya (Ogutu et al. 2016), its interrelation with the use of poisons, often associated with human–wildlife conflict, still remains largely understudied. This knowledge gap is particularly salient in areas of high human–wildlife conflict which also support important vulture populations whose main threat is represented by poisoning, such as Southern Kenya (Ogutu et al. 2016; Virani et al. 2011).

The main aim of this study was to fill the above knowledge gaps by investigating Maasai pastoralists’ attitudes towards vultures and the use of poisons as an adopted practice within conflicting situations between pastoralists and carnivores. The first specific aim was to quantify the attitudes of local Maasai pastoralists towards vultures and other wildlife. Second, quantify the prevalence of poison-use among pastoralists and its distribution in southern Kenya. Third, identify the drivers of poison use in an effort to identify environmental, socio-demographic and policy factors (e.g. awareness of the legislation and regulations) that may promote or discourage the use of poison. The implications of the findings are discussed towards understanding human wildlife conflict more generally, beyond our case study.

## Materials and methods

### Study environment

**Fig. 1:**
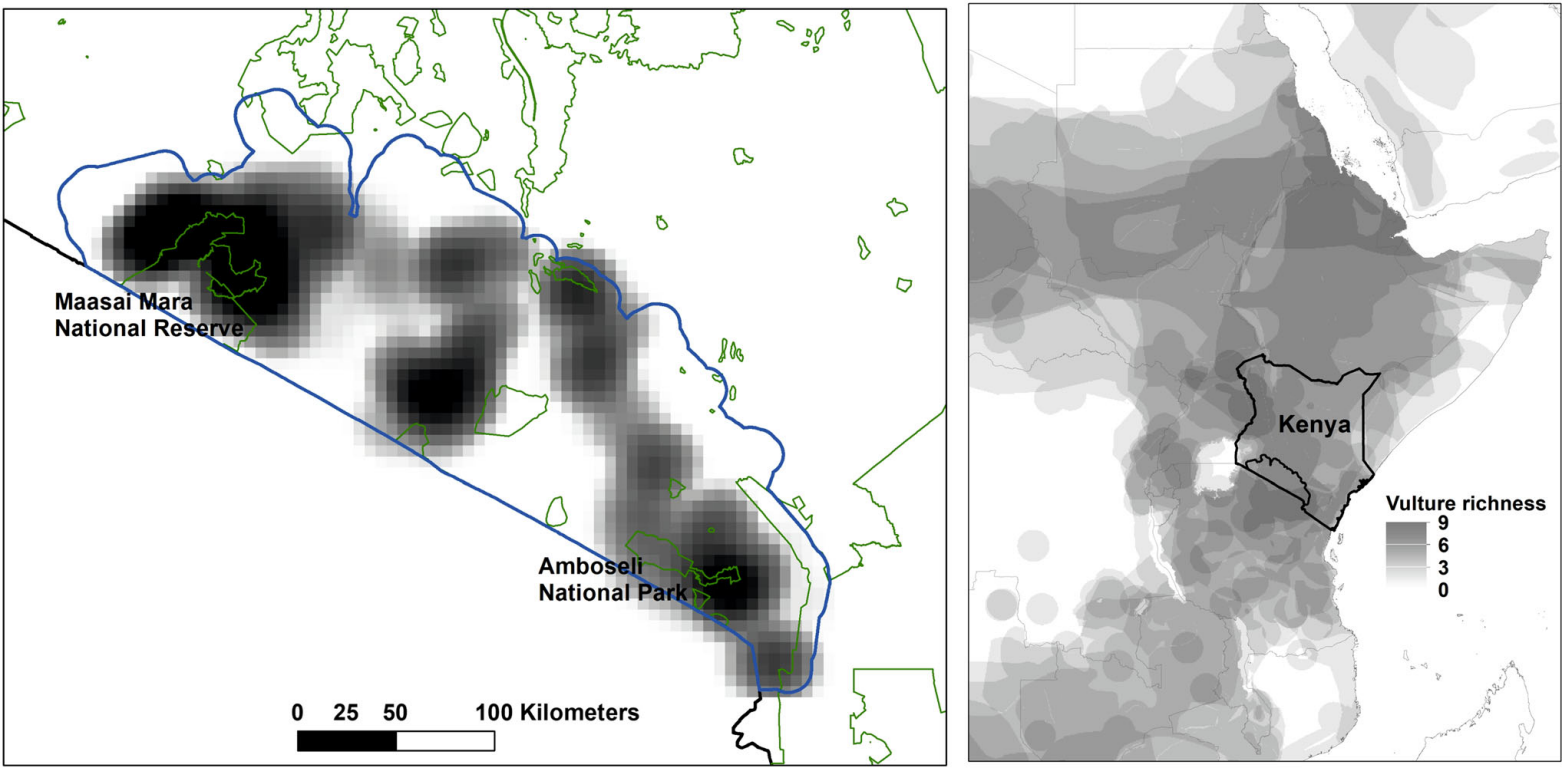
The study region (blue line) in Southern Kenya where the interviews have been conducted (left panel). The black and white gradient areas represent the sampling effort, whereby darker colors depict well-sampled areas, and vice versa for lighter colors. The boundaries of protected areas are shown with green lines. The location of the study region in Southern Kenya is shown in the right panel, with the richness of vulture species shown with the black and white color gradient (darker means higher number of vulture species present)

The study was conducted across the south-western and south-central part of Kenya, stretching along the Tanzania border and surrounding several protected areas (such as the Masaai Mara and Amboseli; Fig. 1). The region is characterized by semiarid conditions, making livestock the predominant source of livelihood, and a critical component of the social settings of pastoralists (Chege et al. 2015). Crop production is instead a marginal activity. The area is dotted with several communal conservancies, areas owned, administered and managed by local communities aimed at wildlife conservation, among others. Under the Wildlife Act, conservancies are acknowledged as a land use, making it an attractive option for land owners and communities as they offer incentives and land resource rights. The region, especially the part bordering the Masaai Mara, supports large herds of migratory wildebeests, as well as other herbivores, and in turn hosts a rich community of predators (such as lions, hyenas and jackals) and also avian scavengers, such as vultures. Six vulture species (*Gyps rueppellii*, *Gyps africanus*, *Torgos tracheliotos*, *Necrosyrtes monachus*, *Trigonoceps occipitalis, Neophron percnopterus*) are found here at different densities (Andreson and Horwitz 1979) and they are all endangered or critically endangered according to IUCN, to the extent that the area represents a global priority hotspot for vulture conservation (Santangeli et al. 2019). The conflict between human and wildlife is pervasive across the study region, involving a range of species and increasing in frequency and intensity over the past decades (Ogutu et al. 2016; Mukeka et al. 2019). Amidst this rising conflicting condition, pastoralists are increasingly making use of poisons in an attempt to eliminate livestock predators. This has resulted in mass mortalities, involving hundreds of vultures, over the recent years (Ogada et al. 2012; Ogada 2014).

### Protocol for data collection

Prevalence of poison-use within the human–carnivore conflict among pastoralist communities was investigated using a questionnaire (see Appendix 1) survey covering the study region and targeted at livestock owners. The survey was conducted between January and August 2018. All interviews were conducted in person by local field assistants who received an extensive 2-days training regarding the protocol for data collection. The field assistants were coordinated locally by E.R. Informed consent to participate in the study was given verbally prior to the interview. Verbal consent was used due to logistic constraints, e.g. the varying literacy level of some of the respondents. If consent was not given, the interview was not taken. Ethics approval for this study was provided by the ethics committee of the University of KwaZulu-Natal (reference number: HSS/0075/018D). Pastoralists were approached by field assistants, each moving across a pre-defined region and selecting respondents that were encountered. This respondent selection was mainly done on an opportunistic basis given logistic constraints, while optimizing the spatial spread of the sampling, broadly following the approach implemented in a recent similar study (Craig et al. 2019). In practice, a field assistant arrived to an area, searched for livestock farmers, and randomly selected one among them, then moved to another location. This was done to ensure spatial coverage of the study area. Overall, because in a pastoral society livestock management, including herding and protection, is a men role, male respondents where more often selected than females. Interviews took 10 to 60 min to complete and were conducted in either Kiswahili or Kimaa language, depending on the respondent’s preference.

### General questions

We designed the questionnaire to include questions on factors that may be associated with a respondent’s propensity to use poison (Appendix 1), as well as questions related to awareness, knowledge and perceptions on vultures and other wildlife. Questions related to basic demography (e.g. age and education level), farming context (e.g. main source of income, type and number of livestock on farm, depredation numbers), attitudes (e.g. towards predators, vultures, and other wildlife), perception of the effectiveness of alternative predator control methods, and knowledge of the Wildlife Act. Attitude and perception questions were framed on a five-point Likert scale of agreement or effectiveness (ranging from strongly disagree to strongly agree or from very ineffective to very effective). We also asked respondents to indicate the percentage of their peers they believed used poison to control predators. The coordinates of the survey point indicating the place where the respondent resides were marked using a hand-held GPS device. From the survey coordinates, we a posteriori calculated the distance of the survey location to the closest protected area (layer obtained from the Word Database on Protected Areas available at: www.protectedplanet.net).

### The list experiment

Using poison to eliminate predators is illegal in Kenya, as in most other countries. Therefore, we used an indirect questioning technique that provides anonymity to respondents and does not require them to directly admit to the illegal behaviour, thereby reducing biases inherent in direct questioning (Nuno and St John 2015). The technique used here, referred to as either the unmatched count technique or list experiment, has been successfully used to quantify illegal hunting prevalence in African communities (Nuno et al. 2013; van Velden et al. 2020) as well as poison use prevalence in South Africa (Brink et al. 2021). Approached respondents were randomly assigned to either a treatment or a control group. Each respondent was presented with a list of behaviours containing four non-sensitive behaviours, but in the case of the treatment group a fifth behaviour, the sensitive one under investigation (poisoning), was added to the list. Respondents were asked to indicate how many of the listed behaviours they have performed during the past year, without indicating the behaviours themselves. All behaviours listed were related to farming practices and one very common and one very rare behaviour was included as recommended to avoid ceiling and floor effects (Blair et al. 2019). The non-sensitive behaviours, or the control items, were livestock herding (common behaviour), crop farming, trading, teaching (the rare behaviour).

### Statistical analysis

Data analyses were performed in R v.3.6.3 (R Core Development Team 2021), using the “list” package, which was specifically designed for analysing list experiments (Blair and Imai 2012). We used the recommended modelling framework provided by Blair and Imai (2012) and recently used by Brink et al. (2021) to estimate poison prevalence and correlates from list experiment data similar to those of this study. We used the Non-linear Least Squares (NLS) estimator and tested our model for assumptions inherent to list experiments, including design and ceiling and floor effects. All predictors were tested for collinearity and highly correlated variables (*r* > 0.7) were excluded from the model. Below we provide a rational for the 10 variables included in the NLS models.

Age was included on the assumption that elder respondents may have fewer alternative methods available for reducing livestock predation and may revert to poisoning as the easiest approach, as discussed in Brink et al. (2021) for South African commercial farmers. The main source of income was considered in the model as respondents whose income largely relates to livestock herding will be more exposed to human–wildlife conflicts and potentially revert to using poison, see also Santangeli et al. (2016).

Two variables related to farming context, such as proportion of predated livestock, and whether predators where considered the main cause of livestock loss, were included as they directly relate to the intensity of human–carnivore conflict and potentially the use of poison thereafter. Similarly, attitudes towards vultures, predators and other wildlife (mainly game and other non-vulture and non-predator species), were included under the assumption that respondents with a more positive attitude would be less inclined to use poison (Santangeli et al. 2016; Craig et al. 2019; Brink et al. 2021). Due to the questionnaire structure, attitudes towards wildlife and predators could be modelled as a continuous variable. The variable used for attitudes towards wildlife was derived from the average of the responses to two questions (wildlife is a valuable resource, and wildlife belongs to conservancies and protected areas) with answers of the type: agree, neutral, disagree. In drawing this average, we coded numerically the answers, from − 1 (answer related to negative attitude), 0 and + 1 (answer related to positive attitudes). The variable for attitude to predators was based on averaging the values, as done above, from answers on three questions: predators are valuable, predators belong to conservancies and protected areas, and predators that kill livestock should be killed. Conversely, attitudes to vultures was used as categorical with two levels, i.e. non-positive (including both negative and indifferent attitudes) or positive attitudes as derived from only one question: It is good to have vultures in this area. Distance to the closest protected areas aimed to assess whether respondents living in the proximity of protected areas may suffer higher losses, and thus revert to poisoning, due to higher densities of carnivores roaming these areas (Santangeli et al. 2016). We included in the model whether the respondent was aware of the Wildlife Act, predicting that those who are aware of the Act would be less incline in using poison. Market accessibility (hereafter accessibility) was extracted at the household location level in GIS using an open access spatial layer (Verburg et al. 2011). The rationale for testing this variable is that pastoralists in less accessible areas may not have easy access to poisons (see e.g. Craig et al. 2019) and may thus not use them as frequently as those living in more accessible areas.

All variables were used as continuous except for four categorical variables with two levels each. These variables include the main cause of livestock loss (other/predators), main income source (other/livestock), and attitude towards vultures (non-positive/positive), and knowledge of the wildlife act (no/yes).

We used the model to predict poisoning probability estimates for each respondent. We then used all the predicted poison probability points in conjunction with the coordinates of the respondents household to interpolate poison probability across the whole study region using the Inverse Square Distance Weighting technique (Neteler and Mitasova 2013). This interpolation thus allowed to visualize the spatial prevalence of poison-use across Southern Kenya, similar to what has been done by Santangeli et al. (2016).

## Results

### Respondents characteristics, livestock herding context and attitudes

A total of 1387 respondents were interviewed, of which 1295 (93.4%) supplied sufficient information to be included in the regression model. These included largely men (69%), with ages ranging from 18 to 33 years (26%), 34–49 (38%) and 50–65 (25%). The most common income source of the respondents household was livestock herding (79%), mainly small (sheep and goat) and large (cattle) livestock. Most respondents (62%) indicated that drought was the primary cause of livestock loss during the preceding year, while predation was indicated by 16% of respondents and was largely attributed (by respondents) to hyenas and lions (83 and 8% of losses, respectively). For the above losses, only 8% of respondents received compensation, which was largely through organized consolation schemes either by conservancies or conservation NGO’s.

**Fig. 2:**
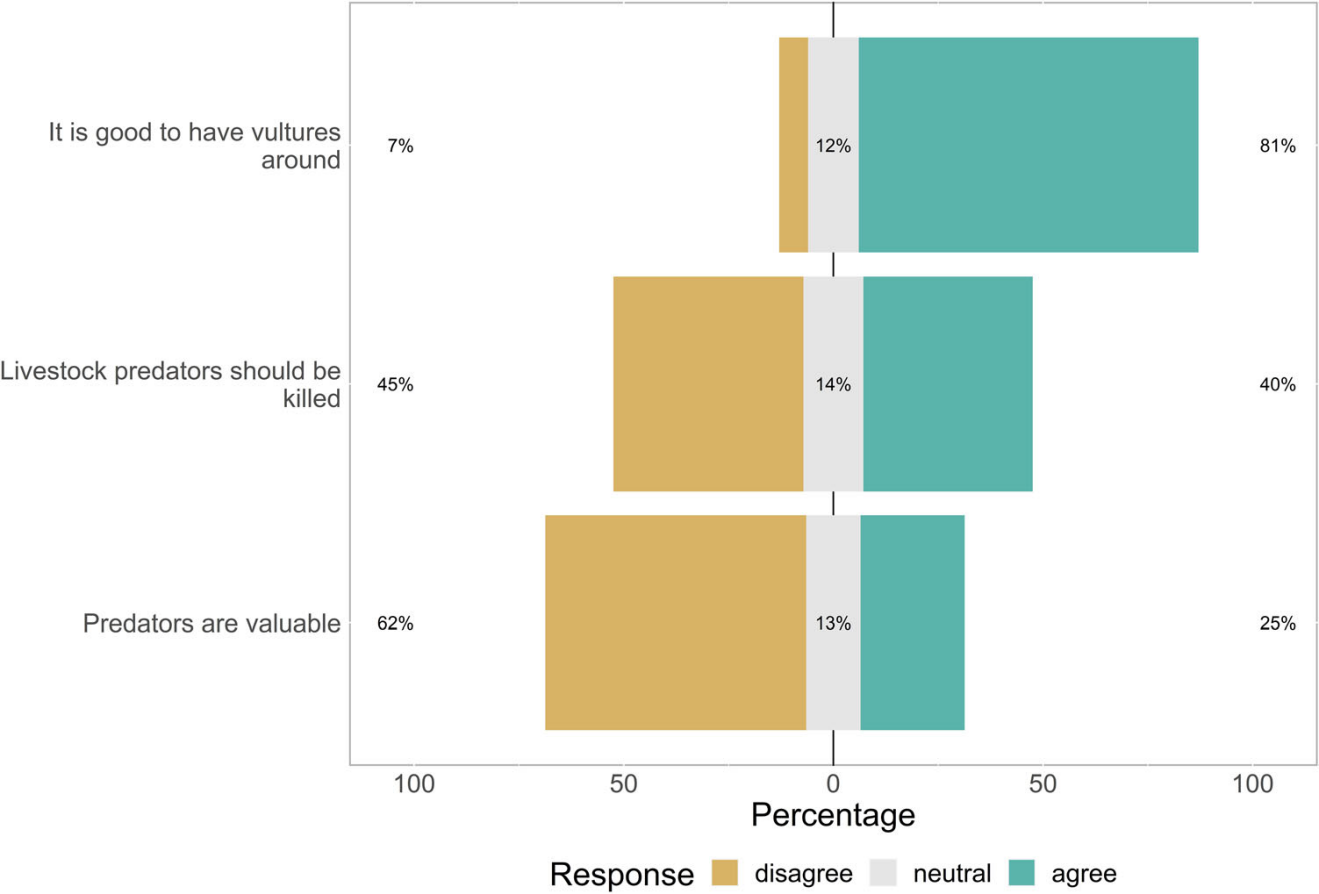
Pastoralists attitudes towards vultures and predators (mainly carnivores) as quantified using a Likert scale from disagree, neutral and agree. The percentages in the figure are relative to each Likert category per question

The vast majority (96%) of respondents indicated that they saw vultures feeding on carcasses. This suggests that they know what a vulture is, and would not mistake it for, e.g., an eagle. Moreover, 91% of respondents reported that vulture populations have declined in their area over the past five years, with 38% of these respondents directly attributing the decline to poisoning. Overall, pastoralists have a largely positive attitude towards vultures (Fig. 2). While views on lethal control of livestock predators are contrasted, the majority of respondents feel that predators are not valuable (Fig. 2).

### Prevalence, correlates and distribution of poison-use

**Fig. 3:**
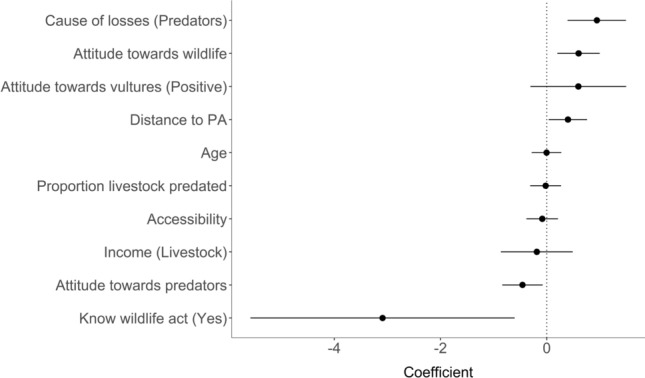
Factors related to the use of poison by pastoralists in Southern Kenya as resulted from the list experiment data. Variable coefficients (dots) and standard error (lines) are derived from multivariate regression models using a non-linear least squares estimator (see methods for more details). The four categorical variables for which results are shown, all have two levels, the one being reported between brackets being the tested one, for which the effect is shown, compared to the reference level. Reference levels were: “Other” (for income and cause of losses), “non-positive” (for attitude towards vulture), and “no” (for know the wildlife act)

There was no evidence for any design effect in our model (*p* ≫ 0.05) and estimates of the proportion of floor and ceiling liars were < 0.2% for both Quasi-Bayesian approximation and maximum likelihood estimates. Based on the list experiment results, an estimated 21.1% (upper and lower 95% confidence interval: 12.7–29.6%) make use of poison to control predators. Moreover, 26% of respondents stated (after direct questioning) that people in their area use poison to kill predators, as this practice is perceived as an effective, easy and safe approach. The model aimed at quantifying the relationship between poison use and a set of factors identified a few predictors that are significantly associated to using poison (Fig. 3). Specifically, pastoralists are more likely to use poison if their main cause of livestock loss is attributed to predators, if they have more positive attitude to wildlife, but more negative to predators, and if they are unaware of the recent wildlife Act of Kenya.

**Fig. 4:**
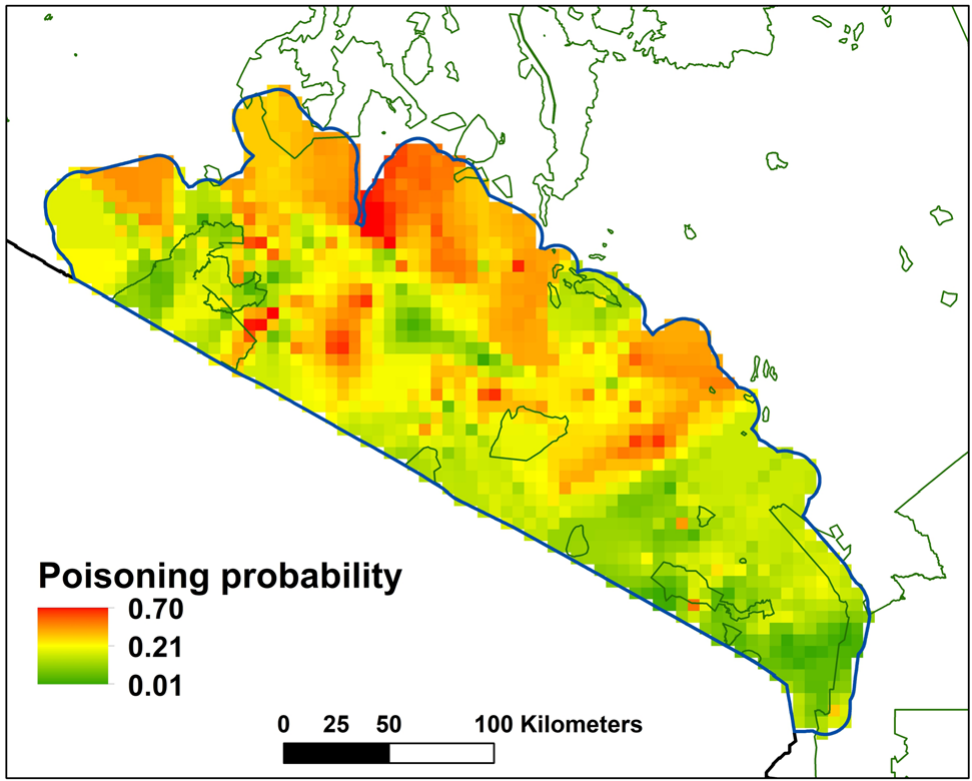
Predicted proportion of poison use probability as derived from the full model (results presented in Fig. 3) based on multivariate regression analysis of the list experiment data and interpolated across the study region. Areas of higher poisoning prevalence (proportion of pastoralists predicted to use poison) are shown with warmer colors, and the boundaries of protected areas within Kenya are shown with green outline

The spatial distribution of predicted poisoning prevalence (Fig. 4) highlights specific local-scale poisoning hotspots, such as those in the central and western side of our study region (e.g. the areas adjacent to the eastern border of the Maasai Mara National Reserve. In these local areas, prevalence of poison use could reach over 50%, basically more than half of the pastoralists using poison to eliminate carnivores. Wide areas of relatively high poisoning prevalence occur in the northern part of the study region, whereas poisoning is relatively less prevalent in the south-eastern areas (Fig. 4), such as around Tsavo National Park.

## Discussion

In this study, we use sensitive and robust questioning techniques to estimate the prevalence of illegal wildlife poisoning among pastoralists in a global biodiversity hotspot and global vulture priority area in Southern Kenya. We show that, while pastoralists have a generally positive attitude towards vultures, but negative attitudes towards other wildlife, over 20% of them use poisons to eliminate predators. Poisoning was most prevalent among pastoralists that suffered livestock losses to predators, that have a more negative attitude towards predators, and also among those that are unaware of the Kenya Wildlife Act of 2013. Moreover, poison use prevalence was highest towards the western part of the study region, around the Masai-Mara National Reserve and towards the north of that.

### Attitudes towards vultures and wildlife

The largely positive attitude towards vultures among pastoralists was expected, because vultures do not represent a threat to people and their livestock, despite very rare cases of predation on injured animals (Lambertucci et al. 2021), and the local pastoralists seem to be aware of that. Instead, vultures are commonly perceived as valuable allies to livestock farmers and owners, as they clean the environment from carcasses, and can act as sentinels. The circling activity of vultures may point livestock owners to places where their animals might have died. This ecosystem service provided by vultures was widely recognized by communal and commercial farmers in Namibia (Santangeli et al. 2016; Craig et al. 2018) as well as in South Africa (Brink et al. 2021). Indeed, the cleaning and sentinel role of vultures were among the most common reasons (50.5% and 14.7%, respectively) given by those respondents with a positive attitude towards vultures (*n* = 1228 responses).

Conversely, attitudes towards predators were largely negative, and are likely shaped by years of conflicts with predators and possibly lack of institutional trust. In the past, coercive conservation measures to prioritize wildlife, and predators, over local wellbeing have caused a marginalization of the Maasai communities in Southern Kenya. These measures have triggered a resentment among local Massaai and generated negative attitudes towards wildlife (Hazzah et al. 2014). This historical aspect, as well as a largely generalized lack of efficient compensation scheme for livestock losses to predators might well explain the negative attitude reported here. These results are also broadly in line with other studies from Southern Africa (e.g. Santangeli et al. 2016; Craig et al. 2018; Brink et al. 2021). Despite the negative attitudes towards predators, polarized views are apparent and similarly split between those in favour or against lethal predator control. This result suggests that, at least half of the respondents are in favour of non-lethal predator control (such as the use of fortified bomas; Sutton et al. 2017) to mitigate the impacts of the existing human–carnivore conflict.

### Poison use prevalence

Prevalence of poison use by Masaai pastoralists in Southern Kenya was very similar to that recently reported among commercial farmers in South Africa and Namibia (Santangeli et al. 2016; Brink et al. 2021). This estimate is also just below the value given by the respondents when asked directly about the prevalence of poisoning among their neighbors (estimated at 26%), suggesting a high consistency of these estimates. While there are no past estimates of poison prevalence that could be compared to the present one, it is likely that this practice was already prevalent, at least to the current levels, during the early 2000s. As an example, a study based on satellite tracked vultures in Southern Kenya estimated a rate as high as 33% annual mortality attributed to poisoning alone (Kendall and Virani 2012), which in turn suggests that poison use must have been relatively common.

### Correlates of poisoning behavior

The main drivers of poison use as resulting from the modeling relate to three broad areas: attitudes (towards predators and towards wildlife in general), the presence of human wildlife conflict, and awareness of the new Kenya Wildlife Act for environmental protection.

Attitudes typically forge individual behaviours, and it is not surprising that those pastoralists with a negative attitude towards predators are more likely to use poisons to resolve a conflict. This, combined with the result that poisoning was more prevalent among those whose main cause of livestock loss was attributed to predators, strongly underscores that poisoning is largely driven by human–carnivore conflicts. A very similar pattern was found among commercial farmers in South Africa and Namibia. Indeed, it is well understood that the large majority of carnivores killed in Kenya, through various means, including poisoning, result from retaliation in response to livestock depredation events (Ontiri et al. 2019). Interestingly, we found that a more positive attitude towards wildlife resulted in more poisoning, which may appear as a counterintuitive result. This result may suggest that while pastoralists may have a positive attitude towards wildlife, they are ready, and will use poisons irrespective, if their livestock is depredated. Indeed, the connection between attitudes and behavior is complex and not always positive, with behavior following attitudes (Ajzen and Fishbein 2005). For example, in conservation, cases where awareness and attitudes do not translate into expected environmental behaviors have been reported (Waylen et al. 2009). However, this unexpected result warrants further and more in-depth investigations which are necessary given the complexity of this system. Such a specific investigation was beyond the scope of this study.

From a conservation policy perspective, the lower prevalence of poison use among those aware of the Wildlife Act is highly relevant. This result suggests that the existence of the legislation, even if just on paper and with poor enforcement, could represent a strong enough deterrent from using poisons. The Wildlife Act explicitly outlines the fines for wildlife crime, such as poisoning, as well as the compensation measures following livestock depredation. Compensation measures, or the so called “consolation payments” (Ogada 2011) because the amount paid is not equivalent to the loss, have shown good success in Kenya, when they are implemented efficiently, which typically happens with specific programs at the local scale (Bauer et al. 2017). These, coupled with the fear of being fined, may result in lowered poison use among those aware of the Wildlife Act, as shown here. Typically, the impact of the law on wildlife crime depends on the perception of the offenders and their attitude towards risk. In general, law enforcement may deter most people who are able to satisfy their basic needs but may not be able to afford to pay fines or be imprisoned. This may be the case for most of those respondents of this study that declared to be aware of the Wildlife Act, and are thereby less prone to take the risk of perpetrating a crime such as poisoning wildlife. For example, a study based at the Bwindi Impenetrable National Park in Uganda, found that law enforcement and associated fear of fines and imprisonment were among the top ranked deterrents against illegal activities by local people (Twinamatsiko et al. 2014). Overall, studies on deterrence to reduce environmental crime have been largely focused on protected areas (Moreto and Gau 2017). Here, we present a rare case where deterrence seems to occur also outside of protected areas, where different socio-economic and environmental drivers may play a role as compared to protected areas.

Overall, it is important to highlight that the social-ecological system studied here is very complex and affected by a multitude of internal and external factors. As such, our study, while aiming at achieving a large and representative coverage of the Maasai pastoralist population, does not allow to delve to the very roots of the system, especially in regards of some of the questions addressed, like the potential deterrent effect of the Wildlife Act. Given the above-mentioned limitations, we encourage further and more in-depth studies that would unveil the intricacies of the human–nature interactions, perceptions and resulting behaviors.

### Distribution of poison use prevalence

High poison use prevalence occurred largely towards the western part of the study region, largely included within the Narok County, an area which supports around 30% of Kenya’s large mammals occurring largely outside of protected areas, such as the Masai Mara (Ogutu et al. 2016). In this region, where the human population has been steadily growing, the human–carnivore conflict has been particularly intense over the past few decades (Ogutu et al. 2016). Here, pastoralists may incur substantial livestock losses to predators (Muriuki et al. 2017; Sutton et al. 2017), which in turn escalates the conflict and result in the use of poisons to eliminate predators.

## Conclusions

We underscore the intimate relationship between human wildlife conflict and the use of poison, with the latter following the former. While a relevant number of pastoralists use poison to eliminate predators, they also share positive attitudes towards vultures. The latter should be leveraged to trigger behavioural change and reduce poison use. This could be achieved not only with punitive disincentives, as part of the Wildlife Act, but also with proactive incentives (such as use of strong corrals to avoid depredation, or compensation to avoid retaliation acts, this latter also part of the Wildlife Act). Overall, the positive impact of the Wildlife Act in reducing poisoning may not only be ascribed to a deterrent effect, but also to the enhanced legitimacy of the Wildlife Act. This recognizes and encourages the practice of wildlife conservation for every individual as a form of land use, e.g. through the establishment of communal conservancies, areas that are managed by people and for wildlife in a sustainable way (Naidoo et al. 2016). Ultimately, while the apparent impact of the Wildlife Act on reducing poisoning is encouraging, it is still far from stopping this illegal practice. Intensive efforts, focusing on the most critical areas as identified in this study, should be urgently put in place. Such efforts could focus on increasing the awareness of the Wildlife Act among local people, and the further implementation of proactive and compensatory measures that have been found to be effective (such as corrals or compensation). Such efforts would likely benefit not only the declining vulture populations, but also the wider wildlife sharing the landscape with people.

## Supplementary Information

Below is the link to the electronic supplementary material.

Supplementary file1 (PDF 135 kb)

## Data Availability

All data used for the poison modeling is archived in Figshare (https://doi.org/10.6084/m9.figshare.17197784) without information that could refer to individual respondents, such as the coordinates.
