# Supplementary material for: Awareness of environmental legislation as a deterrent for wildlife crime: A case with Masaai pastoralists, poison use and the Kenya Wildlife Act
Source: Ambio. 2022 Jan 25;51(7):1632–42. doi: 10.1007/s13280-021-01695-8 (PMC9110623; doi:10.1007/s13280-021-01695-8)
Supplement: Supplementary file 1 — Supplementary file1 (PDF 135 kb) [file 13280_2021_1695_MOESM1_ESM.pdf]

Ambio

Electronic Supplementary Material
This supplementary material has not been peer reviewed

Title: **Awareness of environmental legislation as a deterrent for wildlife crime – a case with Masaai pastoralists, poison use and the Kenya Wildlife Act**

Authors: Zahra Didarali, Timothy Kuiper, Christiaan W. Brink, Ralph Buij, Munir Virani, Eric O. Reason, Andrea Santangeli

Appendix 1

Questionnaire for list experiment with livestock farmers

Consent given ________

Questionnaire number_________________ **Interviewer**_____________________

Date____________ **Time started**_____________________

Village___________________

Coordinates S _______________ E ____________

18-33	34-49	50-65	65-80	80+

1) Gender: Male______ Female__________ **2)** Age

2) What is your household's main source of income? __________________

Livestock

3) In the last 12 months what is the main cause/s of livestock loss? (rank)

1.
2.
3.

4) Livestock numbers and losses to predators (in the last 12 months)?

Type of Livestock	Number	N lost	Approximate cost of lost livestock	N lost to Predators	Compensation (Yes/No)?
Cattle					
Sheep					
Goats					
Donkeys					
Chicken					

5) Which predator/s is most problematic? (rank)

1.
2.
3.
4.

Wildlife and predators

6) I am going to say a statement tell me if you agree, disagree or have no opinion on this

No.	Statement	Agree	Neutral	Disagree
**a)**	Wildlife is a valuable resource to my community			
**b)**	Wildlife belong in Protected areas and conservancies and not in the community land			
**c)**	Predators are a valuable resource to my community			
**d)**	Predators should be confined in conservancies or other protected areas			
**e)**	Predators that kill livestock should be killed			

Vultures

I am going to say a statement tell me if you agree, disagree or have no opinion on this

7) It is good to have vultures in this area

Agree	Neutral	Disagree

8) Why do you agree/disagree with this?____

9) What is the main thing that you see vultures eating? ______________________________________

10) In the last five years have the number of **vultures** in this area?

Decreased: ______ Increased:____ Stayed the same:____

Why do you think this is? ____

##

Use the unmatched-count technique now to assess poison use by the respondent

Toss a coin if tails (treatment), heads (control), give card. Ask how many behaviours are relevant to them. Never ask which behaviours, this is sensitive information we must not ask and know.

Control or Treatment: C_____ T______ Number of behaviours relevant: _______

##

Other farmers in your ward (peers behaviour):

11) In your ward what do you think are the most popular method/s used by the community to kill predators? (rank)

1.
2.
3.

12) Do people in your ward use poison to kill predators? Yes_______ No________

13) Do you know about the new wildlife conservation Act 2013?

Yes…………. No…………………..

a) If yes what do you know about the new Act? ____

b) What do you like about the new Wildlife Act?

____

c) What do you dislike about the new Wildlife Act?

____

Time finished__________
